# Inhibitory postsynaptic density from the lens of phase separation

**DOI:** 10.1093/oons/kvac003

**Published:** 2022-05-04

**Authors:** Guanhua Bai, Mingjie Zhang

**Affiliations:** School of Life Sciences, Southern University of Science and Technology, Shenzhen 518055, China; School of Life Sciences, Southern University of Science and Technology, Shenzhen 518055, China; Greater Bay Biomedical Innocenter, Shenzhen Bay Laboratory, Shenzhen 518036, China

**Keywords:** phase separation, biomolecular condensate, postsynaptic density, excitatory synapse, inhibitory synapse, gephyrin

## Abstract

To faithfully transmit and decode signals released from presynaptic termini, postsynaptic compartments of neuronal synapses deploy hundreds of various proteins. In addition to distinct sets of proteins, excitatory and inhibitory postsynaptic apparatuses display very different organization features and regulatory properties. Decades of extensive studies have generated a wealth of knowledge on the molecular composition, assembly architecture and activity-dependent regulatory mechanisms of excitatory postsynaptic compartments. In comparison, our understanding of the inhibitory postsynaptic apparatus trails behind. Recent studies have demonstrated that phase separation is a new paradigm underlying the formation and plasticity of both excitatory and inhibitory postsynaptic molecular assemblies. In this review, we discuss molecular composition, organizational and regulatory features of inhibitory postsynaptic densities through the lens of the phase separation concept and in comparison with the excitatory postsynaptic densities.

## INTRODUCTION

**Figure 1:**
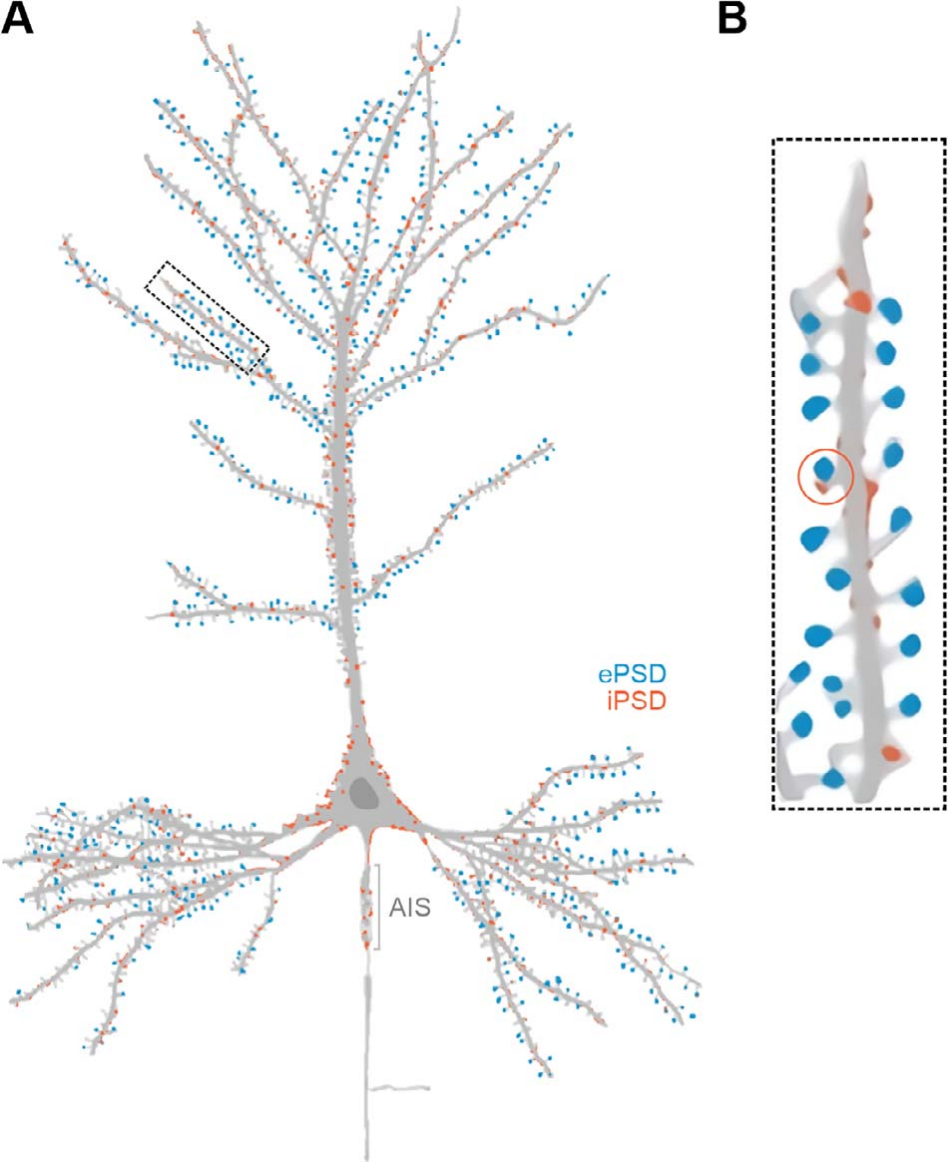
**Distributions of excitatory and inhibitory synapses.** Schematic drawing of a typical pyramidal neuron with excitatory (blue) and inhibitory (red) postsynaptic compartments labeled as colored dots. (A) Excitatory synapses are localized on dendritic spines. The majority of inhibitory synapses are located at the dendritic shaft adjacent to excitatory synapses. There are also many inhibitory synapses on cell soma and axon initial segments (AIS). (B) Note that a small portion of inhibitory synapses (e.g. the one indicated by a circle) can also form on dendritic spines adjacent to excitatory synapses.

Excitatory and inhibitory synapses differ remarkably in morphology, molecular composition and organization. Under electron microscope (EM), the two classes of synapses were identified as type I or asymmetric synapses characterized by an apparent postsynaptic density (PSD) on dendritic spines and type II or symmetric synapses without evident thickening and usually found on cell bodies or dendritic shafts (Fig. 1). Excitatory PSDs (ePSDs) are highly stable and can be biochemically purified. In addition to glutamate receptors, hundreds of other proteins have been identified as consensus ePSD proteins, including cell adhesion molecules, scaffold proteins, signaling enzymes and cytoskeleton elements [1]. Super-resolution imaging further revealed that proteins in the ePSD display a laminar distribution along the axodendritic axis [2]. In sharp contrast, much less is known regarding the molecular composition of inhibitory PSDs (iPSDs), largely due to the much more dynamic nature of inhibitory synapses. The development of modern proteomic techniques began to offer opportunities in identifying proteins forming iPSDs [3]. An overarching feature for both ePSD and iPSD is that numerous proteins appear to be able to autonomously assemble into elaborate molecular networks with high concentrations of proteins beneath the postsynaptic plasma membranes. Extensive studies in the past have provided compelling evidence showing that both the sizes and dynamics of PSDs in both excitatory and inhibitory synapses are highly correlated with synaptic activity. A larger PSD contains more neurotransmitter receptors and thus tends to be a stronger synapse. Neurotransmitter receptors within a PSD can further undergo activity-dependent redistribution to form nanodomains/nanoclusters to enhance synaptic potentiation [4–7]. A series of recent studies have provided evidence showing that such highly condensed PSD molecular assemblies in both excitatory and inhibitory synapses are formed via liquid–liquid phase separation ([8–11]; also see [12–14] for reviews). Additionally, phase separation has also been implicated in the dense active zone complex assembly [15], reserve pool synaptic vesicle clustering [16] and readily releasable vesicle docking on active zones [17].

In this review, we present our current understanding of the molecular organizations of postsynaptic assemblies through the lens of phase separation. We focus our description on the iPSD and in a comparative view with the ePSD.

## MOLECULAR ORGANIZATION OF POSTSYNAPTIC DENSITIES

### The molecular architecture of the ePSD

**Figure 2:**
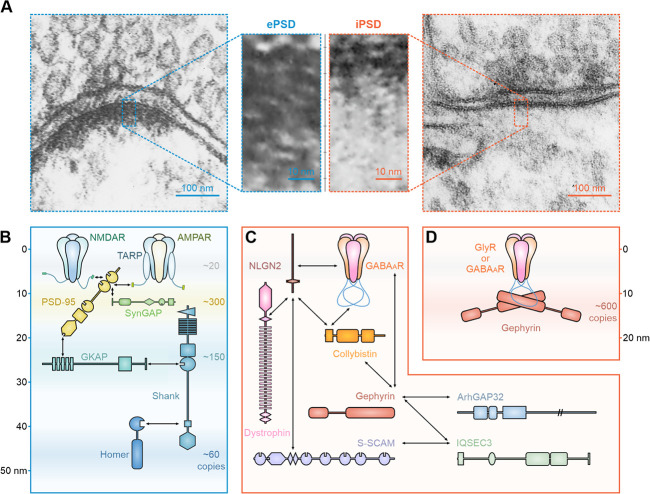
**Distinct molecular organizations of the ePSD and the iPSD.** (A) EM images of an excitatory (left blue boxes) and an inhibitory (right red boxes) synapse. Zoom-in views of corresponding boxed areas show the thick ePSD and thin iPSD. Images are adapted from SynapseWeb (https://synapseweb.clm.utexas.edu/). (B) Domain architecture and interaction network of the major ePSD proteins. The diagram was drawn according to the supramolecular organization of the ePSD. The location of each protein is proportional to the mean position derived from immunogold staining. The interactions between pairs of proteins are indicated using arrows. The axodendritic axis is indicated on the left with the postsynaptic plasma membrane as the starting position (0 nm on the axis). The copy number of each component per average ePSD (‘stoichiometry’) is shown on the right. (C) Domain architecture and interaction network of known iPSD proteins. (D) Multivalent interactions between inhibitory receptors and gephyrin. The supramolecular organization of the iPSD is drawn according to the recent EM tomography study [24].

The apparent electron-dense thickening beneath the postsynaptic membrane under EM is the hallmark of excitatory synapses. Typically, the ePSD has a disc-like shape with 200–800 nm in diameter and 20–50 nm in thickness (Fig. 2A) [18]. Major scaffold proteins interacting with each other form a layered organization along the axodendritic axis, serving to cluster glutamate receptors on the postsynaptic plasma membranes (Fig. 2B). Various biochemical and biophysical studies have provided an estimation of absolute copy numbers of key proteins in the ePSD [19–22]. For example, calcium/calmodulin-dependent protein kinase II (CaMKII) is the most abundant protein (~5600 copies of monomer or ~450 copies of the holoenzyme) in the ePSD functioning as a catalytic enzyme as well as a scaffold protein critical for synaptic plasticity. The PSD-95 family of membrane-associated guanylate kinase (MAGUK) proteins is also prevalent (~400 copies), with PSD-95 as the dominant member (~300 copies) in the ePSD. Other scaffold proteins, including guanylate kinase-associated protein (GKAP or SAPAP, ~150 copies), SH3 and multiple ankyrin repeat domains proteins (Shank, ~150 copies) and Homer (~60 copies) family proteins, also present in high abundance. Through specific intra- and inter-molecular interactions, these multidomain scaffold proteins form inter-connected molecular assemblies capable of clustering glutamate receptors and concentrating signaling enzymes (Fig. 2B) (see [12–14] for reviews).

### Gephyrin, a master organizer in the iPSD

The iPSD has a sheet-like structure with a surface area of ~0.05 μm^2^ on average and a thickness of ~12 nm (Fig. 2A) [23, 24]. Because the iPSD cannot be readily isolated, the detailed molecular composition and organization of the iPSD are much less understood compared to the ePSD. Glycine receptors (GlyRs) and GABA_A_ receptors (GABA_A_Rs) are the major neurotransmitter receptors of inhibitory synapses. Unlike the elaborate scaffold machinery in the ePSD, gephyrin is the only well-recognized scaffold protein in the iPSD. A quantitative nanoscopic imaging study revealed that each inhibitory synapse contains ~600 molecules of gephyrin in GlyR-containing spinal cord synapses and ~130–190 copies of gephyrin in GABA_A_R-containing synapses [23]. Interestingly, GlyRs or GABA_A_Rs are with a density similar to that of gephyrin in the iPSDs, indicating that the gephyrin scaffold is highly occupied by the receptors.

Gephyrin is the master iPSD organizer capable of linking transmembrane receptors with downstream signaling proteins for inhibitory synaptic transmission (Fig. 2C and D). It was initially identified as a 93-kDa GlyR-associated protein cofractionated with tubulin [25, 26], thus was thought to ‘bridge’ GlyRs with the synaptic microtubules [27]. Immunostaining studies revealed a primary synaptic localization of gephyrin with both GlyRs and GABA_A_Rs, but not with glutamate receptors, in neurons [28, 29]. Gephyrin is remarkably conserved in vertebrates. Its two structured domains, N-terminal G-domain and C-terminal E-domain, are homologous to *Escherichia coli* molybdenum cofactor (Moco)-synthesizing enzymes MogA and MoeA, respectively [30]. Crystal structures of the G-domain trimer and the E-domain dimer, as well as several complexes of the E-domain with its binding partners, have been solved (see [31] for review). The oligomeric states of the G- and E-domains suggest a hexagonal lattice model for high-order gephyrin assemblies [32]. However, the proposed lattice structure has never been experimentally observed, likely due to the high flexibility of its central (C) domain [33], which has ~150 amino acid residues.

### **GlyRs and GABA**_**A**_**Rs are clustered at iPSDs with gephyrin**

The most critical role of gephyrin is perhaps to cluster GlyRs or GABA_A_Rs at inhibitory synapses. Initial mapping studies found that the cytoplasmic loop connecting transmembrane helices 3&4 (TM3–4) of the GlyR β subunit is responsible for binding to gephyrin [34]. Later structural studies confirmed that a short peptide from TM3–4 of the GlyR β subunit binds to the gephyrin E-domain [35]. Mutations of key residues in gephyrin disrupting its interaction with GlyR led to increased mobilities of the receptors both within and outside synapses [36]. Deleting gephyrin in cultured spinal neurons further prevented GlyR from clustering [28].

GlyRs belong to the ligand-gated, pentameric chloride channel superfamily and are predominantly expressed in spinal cords and brain stems. The composition of GlyRs varies in different regions and developmental stages, each composed of various combinations of four α subunit isoforms (α1–α4) and one β subunit. Both α and β subunits are Cys-loop family members and share homologous sequences and common structural architectures. GlyRs in adult spinal neurons are generally heteropentamers composed of three α1 and two β subunits, whereas most GlyRs in developing neurons are composed of α subunits only [37, 38]. The GlyR β subunit alone cannot form functional GlyRs and mainly acts as structural subunits but is capable of participating in agonist binding. Because postsynaptic clustering of GlyRs is mediated by the β subunit binding to gephyrin, homomeric GlyRs formed by α subunits exhibit diffused extrasynaptic localization [39].

Most of the fast-neuronal inhibitions in the brain are mediated by GABA_A_Rs, which are also chloride-selective pentameric ligand-gated ion channels. There are 19 subunits from eight subunit classes (α, β, γ, δ, ε, π, ρ and θ). Typically, functional GABA_A_Rs are composed of two α subunits, two β subunits and a single γ or δ subunit. Thus, the possible combinations of GABA_A_Rs are very large. The α1–3, β2–3 and γ2 subunits are enriched in PSDs, whereas the α4–6 and δ subunits are located at extrasynaptic sites [40]. Several subunits (α1, α2, α3, β2 and β3) of GABA_A_Rs have also been found to bind to gephyrin [41–44]. Although with much lower (several hundred folds or more) affinities when compared to the GlyR/gephyrin interaction, gephyrin is essential for the GABA_A_R clustering and synaptic transmission of the majority of GABAergic synapses [36, 45]. The sheer diversity of GABA_A_Rs has imposed technical challenges for understanding the molecular mechanisms of GABA_A_R-mediated synaptic inhibitions. For example, it is not well understood what the subunit compositions of GABA_A_Rs in synapses formed by each inhibitory interneuron are. To make the matter more complicated, the types of interneurons in the animal brain appear to be very diverse and not well characterized.

### Molecular composition and heterogeneity of the iPSD

A recent elegant proteomic study using *in vivo* proximity-dependent biotin identification (BioID) technique identified ~180 proteins enriched in the iPSD of juvenile mice [3]. The study significantly expanded the list of proteins existing in the iPSD. Additional proteomic-based profiling, as well as quantification of iPSD proteins for different ages of mice, will be very valuable. Additionally, the composition of iPSD proteins identified from the BioID approach represents an ensemble average of diverse inhibitory synapses existing in the mice brain. It would be ideal if methods were developed for isolation and quantification of iPSD proteins of each specific type of inhibitory synapse.

Many iPSD proteins are known as direct gephyrin binders and with diverse functions (Fig. 2C). Neuroligin-2 (NLGN2) is the first identified inhibitory synapse-specific cell adhesion molecule and is also the only cell adhesion molecule reported to interact with gephyrin. Thus, NLGN2 is of considerable interest in inhibitory synapse formation and function. Intriguingly, although NLGN2 is known to exist in most GABAergic synapses, deletion of NLGN2 only perturbed gephyrin clustering in perisomatic sites [46]. NLGN2 can also bind to and activate collybistin (also known as Arhgef9), another gephyrin-binding protein abundantly existing in iPSDs [46, 47]. Collybistin is a neuron-specific guanine exchange factor and activates small GTPases of the Rho family. It is also one of a few well-known inhibitory synaptic proteins present at both glycinergic and GABAergic postsynapses. A recent study revealed that the α2 subunit of GABA_A_Rs could directly bind to the collybistin-SH3 domain and induce collybistin-mediated translocation of gephyrin to the postsynaptic membranes [48]. However, deletion of collybistin did not seem to affect synaptic GlyRs clustering and the formation of a substantial subset of GABAergic synapses, implying that collybistin is not an obligatory component for the iPSD formation [49–51]. Signaling enzymes, such as IQSEC3 and ArhGAP32, have also been reported to localize exclusively at inhibitory postsynapses and interact with gephyrin [3], although with unknown molecular mechanisms.

The stability of the iPSD depends on the integrity of two cytoskeletal systems, microtubules and actin filaments [52, 53]. It was observed that GlyRs could form small intracellular clusters and are co-transported with gephyrin along microtubules [54]. Mechanistically, gephyrin can associate with microtubules via binding to the 8-kDa dynein light chains (Dlc1/2) of the dynein complex or via directly binding to KIF5 kinesin motor [54–57]. As for linking with the actin filaments, gephyrin has been shown to form complexes with actin polymerization regulators, including profilin and the elongation factor 1A (eEF1A), or to interact with microfilament adaptors such as neuronal vasodilator-stimulated phosphoprotein (VASP) orthologs Mena and Evl [3]. Single-particle tracking analysis indicated that interactions with components of the actin cytoskeleton might impact synaptic localization and lateral dynamics of gephyrin–GlyR clusters [58]. The role of cytoskeletons in postsynaptic GABAergic synapse formation is less clear.

Compared to glycinergic synapses, GABAergic synapses exhibit a higher level of variability in the molecular composition and functional properties. A subset of GABA_A_R clusters in specific neurons or subcellular compartments is unaffected even when gephyrin is absent [59], indicating existence of compensatory or redundant mechanisms for the iPSD formation. Additional candidates, such as the dystrophin–glycoprotein complex (DGC), have been proposed to mediate GABAergic synapse formation. DGC is required to stabilize GABA_A_Rs, but not gephyrin, in perisomatic synapses [60]. DGC is also involved in the clustering of scaffold proteins (S-SCAM; also known as MAGI2), adhesion molecules (such as NLGNs) and signaling enzymes (including IQSEC3) at the inhibitory synapses [61]. As mentioned, S-SCAM, another MAGUK family scaffold protein, is also presented in the iPSD. S-SCAM is better characterized as a binding partner for ePSD proteins, including GKAP, TARP (transmembrane AMPAR regulatory proteins), NMDARs and NLGN1. Whether S-SCAM functions as another scaffold critical for inhibitory synapse formation and/or regulation remains unknown. Recently, two newly identified proteins, InSyn1 and InSyn2 (inhibitory synapse protein 1 and 2), were added to the list of possible iPSD scaffolds [3]. Both proteins are highly enriched and have extensive interactions with core components of the iPSD. InSyn1, in addition to binding to gephyrin, can directly interact with the DGC complex, thus regulating DGC-mediated GABAergic synapse formation [62]. Whether, and if yes, how InSyn1 and InSyn2 regulate other inhibitory synapse formation and function needs further investigation.

## PHASE SEPARATION UNDERLIES THE FORMATION OF POSTSYNAPTIC ASSEMBLIES

### Phase-separation-mediated ePSD organization

Cells are highly compartmentalized. In addition to the classical membrane-enclosed cellular organelles, formation of membrane-less compartments is increasingly recognized as a general strategy for diverse cellular processes, including cell signaling, cell polarity establishment and maintenance, cell and organ development, cell survival and aging [63, 64]. These membrane-less compartments can form via phase separation, a physical process in which a homogeneous aqueous molecular mixture spontaneously demixes into two separated phases: a dilute phase and a condensed phase. Because there is no physical boundary between the two phases, molecules in the two phases can undergo constant exchanges. But the net flux of molecules between the two phases is zero once the phase separation process has reached its equilibrium. Thus, phase separation provides a physical means for creating a distinct type of cellular organelles with respect to the membrane-delimited organelles. Compared to membrane-based organelles, such membrane-less organelles (or organelles not enclosed by lipid membranes to be more inclusive) display many unique features including but not limited to formation/dispersion mechanism and kinetics, molecular exchanges between organelles and surroundings, responding to regulatory inputs, orchestrating catalytic reactions, tuning properties of molecular interactions, etc.

Neurons take cellular compartmentalization to an extreme due to their high polarities and elaborate morphologies. Specialized structures of presynaptic boutons and postsynaptic dendritic processes pose logistic challenges for biomolecules and their complexes to be properly transported to and then enriched in their respective places. The physically compartmentalized structures of presynaptic boutons and dendritic spines, and more importantly, the directionality of the information flow along the axodendritic axis, create geometric constraints to the positioning of molecular apparatuses in both presynaptic boutons and postsynaptic spine protrusions.

Emerging evidence shows that the formation of the highly condensed ePSD is driven by phase separation. Initially, it was observed that two abundant proteins in the ePSD, PSD-95 and SynGAP, underwent phase separation when purified proteins were mixed in test tubes [8]. Biochemical studies revealed that this process was mediated by both the high-affinity interaction between the two proteins and the homo-trimerization of SynGAP. Disruption of their multivalent interactions abolished condensate formation and reduced the synaptic enrichment of SynGAP in cultured hippocampal neurons. Conversely, increasing the valency by introducing the N-terminal two PDZ domains of PSD-95 (i.e. using the full-length PSD-95 instead of the PDZ3-SH3-GK tandem in the initial work) further promoted the condensate formation of the complex [9].

The proposed phase separation-mediated ePSD assembly model was then extensively tested using *in vitro* reconstitution approaches [9, 10]. The major scaffold proteins, PSD-95, GKAP, Shank and Homer (Fig. 2B), could form phase-separated condensates when mixed. The condensates formed by the ePSD scaffolds could recruit SynGAP as well as cluster glutamate receptors in solution and on lipid membrane bilayers. The threshold concentration for the ePSD mixture to undergo phase separation was far below their physiological concentrations at synapses. Again, specific and multivalent interactions between scaffold proteins governed the condensate formation. These multidomain proteins are either capable of self-multimerizing or specifically interacting with multiple target proteins. Importantly, the reconstituted ePSD condensates consisted of many features of ePSD observed in neurons: it is a self-organized molecular condensate not enclosed by lipid membranes; proteins within the ePSD condensate can dynamically exchange with their counterparts in dilute solution (i.e. spine cytoplasm); scaffold proteins show layered organization with the upper layer of receptors and PSD-95 being linked with a lower layer of the Shank and Homer by GKAP; the ePSD condensate assembly could be modulated through component changes in the network, which partially mimics synaptic plasticity during neuronal activity changes; other synaptic molecules can be selectively enriched or excluded, etc.

### **Gephyrin undergoes phase separation with GlyRs and GABA**_**A**_**Rs**

Different from excitatory synapses, which reside on specialized protrusions from postsynaptic dendrites, most inhibitory synapses are localized at cell soma or dendritic shafts lacking spatial confinements (Fig. 1). Nonetheless, inhibitory synapses also contain iPSDs beneath the postsynaptic membrane. A high density of neurotransmitter receptors is juxtaposed to presynaptic vesicle release sites, although iPSD is with a sheet-like structure and thus much thinner than ePSD [65]. To form discrete dense clusters containing receptors and the gephyrin scaffold amidst the surface of synaptic plasma membranes, an autonomous self-assembly mechanism analogous to ePSD assembly seems necessary for iPSD.

We recently directly tested whether iPSD complexes composed of gephyrin and GlyR or GABA_A_R might form condensed assembly via phase separation [11]. When mixing the dimeric E-domain of gephyrin and the cytoplasmic TM3–4 loop of GlyR or GABA_A_R, their complex spontaneously assembled into highly condensed clusters at physiological buffer conditions. The threshold concentrations of the proteins for the condensates to form were as low as several micromolar in solution and several dozen nanomolar on supported membrane bilayers, suggesting that the GlyR/gephyrin and GABA_A_R/gephyrin complexes can autonomously form dense clusters via phase separation in living neurons. Both the core hydrophobic interaction, which is visualized in the crystal structure of the GlyR peptide and the gephyrin E-domain complex [66], and the weak charge–charge interaction between positively charged residues upstream of the hydrophobic core of GlyR or GABA_A_R and a negative charge surface on the gephyrin E-domain are required for the receptor and gephyrin mixture to phase separate.

Both the valency and affinities of the interactions between gephyrin and receptors are critical for their phase separation. Biochemical manipulations decreasing their binding affinity or lowering the valency of the interaction weakened the phase separation. Interestingly, though GABA_A_R showed a much lower affinity than GlyR in binding to gephyrin, it exhibited a similar concentration threshold to phase separate with gephyrin when compared with GlyR. Thus, it appears that certain residues in the TM3–4 loop of GABA_A_R also contribute to the phase separation upon binding to gephyrin.

It has been a mystery how phosphorylation of the gephyrin C-domain may regulate the clustering of receptors, as only the E-domain of gephyrin is involved in binding to the receptors. It turned out that phosphorylation of different residues in the C-domain can bidirectionally modulate the phase separation capacity of gephyrin. For example, phosphorylation of Ser268 or Ser270 was found to weaken the phase separation of gephyrin with receptors. In contrast, phosphorylation of Ser305 could enhance the phase separation of gephyrin with receptors. Mechanistically, different segments within the C-domain can interact, albeit rather weakly, with the E-domain of gephyrin, thus modulating the phase separation property of gephyrin. Phosphorylation of Ser residues in different segments of the C-domain had the opposite role in modulating the binding between the C- and E-domains and thus differentially impact the clustering of gephyrin with receptors [11].

Additionally, phase separation of the gephyrin/receptor complex could also be regulated by other iPSD proteins. For example, Dlc1/2 can specifically bind to two short sequences within the C-domain of gephyrin. The binding of Dlc1/2 to gephyrin significantly enhances the phase separation capacity of gephyrin via two distinct mechanisms: Dlc1/2 binding-induced dimerization (thus increasing the gephyrin/receptor complex valency) and release of E-domain auto-inhibition by the C-domain. The example of Dlc1/2 suggests that other gephyrin binding proteins, including some of the ones identified in the recent proteomic study [3], may also modulate the phase separation capacity of gephyrin and consequently regulate iPSD formation.

Collectively, instead of forming an elaborate and highly stable scaffold protein network by multiple scaffold proteins in the ePSD, the iPSD uses gephyrin as the key scaffold, if not the sole one, to orchestrate the clustering of GlyR or GABA_A_R through phase separation. Accordingly, gephyrin becomes to be the main hub for regulating iPSD activities. Whereas in ePSD, every major scaffold protein can serve as a target for regulation.

## PROPERTIES AND DYNAMICS OF IPSD AND EPSD CONDENSATES

### Receptors serve as clients in the ePSD, but as drivers in the iPSD

Neurotransmitter receptors constantly switch within and outside the PSD during synaptogenesis and synaptic plasticity. Single-particle tracking analysis revealed that receptors displayed rapid Brownian diffusions on the extrasynaptic membranes with relatively homogeneous diffusion coefficients. Whereas different types of receptors exhibit different levels of confined motions at synapses. In excitatory synapses, α-amino-3-hydroxy-5-methyl-4-isoxazolepropionic acid receptors (AMPARs) are more mobile than N-methyl-D-aspartate receptors (NMDARs) [67, 68]. The amount of synaptic AMPARs could be rapidly modulated by neuronal activities. For inhibitory synapses, GABA_A_Rs tend to diffuse faster and to escape more easily from synaptic sites than GlyRs [69]. The residence time of receptors within PSDs likely reflects their associated scaffold protein networks and cytoskeletons. The ePSD condensates are composed of a large set of scaffold proteins forming intricate molecular networks, whereas the iPSD condensates are chiefly formed by the single scaffold protein gephyrin. Accordingly, NMDARs and AMPARs in the ePSD are less mobile than GlyRs and GABA_A_Rs in the iPSD.

The dynamic properties of excitatory and inhibitory neurotransmitter receptors observed in synapses in neurons are well recapitulated in the *in vitro* reconstituted systems. The ePSD condensate formation does not depend on NMDAR or AMPAR but instead is driven by the specific and multivalent interactions between the scaffold proteins [9]. Thus, NMDARs and AMPARs are regarded as ‘clients’ that can be passively enriched and clustered in the ePSD condensates. As ‘clients’, the copy number of NMDARs or AMPARs can vary but are always less than the scaffold slots (e.g. PSD-95) in the ePSD. In inhibitory synapses, postsynaptic clustering of GlyRs and GABA_A_Rs depends on gephyrin. Reciprocally, the synaptic restriction of gephyrin also depends on its interaction with the receptors, as targeted deletions of GlyR or different subunits of GABA_A_R lead to impairments of synaptic clustering of gephyrin. [61, 70]. Additionally, neurotransmitter receptors and gephyrin often undergo synchronized alterations both in their numbers and diffusion properties upon synaptic activity changes [23]. Therefore, GlyRs or GABA_A_Rs, together with gephyrin, function as ‘drivers’ in the formation of iPSD condensates. As obligatory components that drive the formation of the iPSD condensates, the stoichiometry of the inhibitory receptors to the gephyrin scaffold is rather stable and matches with the number of the binding sites available in gephyrin (i.e. ~1:1 stoichiometry) as observed in the synapse *in vivo* and in the reconstituted iPSD condensates *in vitro* [11, 23].

### The laminar ePSD and planar iPSD structures as results of distinct postsynaptic scaffold assemblies

The distinct PSD shapes are one of the hallmarks to distinguish excitatory and inhibitory synapses under EM. A typical ePSD exhibits a thick and heterogenous disc-like electron density, whereas the iPSD shows a much thinner sheet-like structure (Fig. 2A). The ePSD is a multimolecular assembly organized by different families of scaffold proteins. These scaffold proteins interact with each other forming a layered molecular organization (Fig. 2B) [2, 71–73]. When mixed, these proteins together form highly concentrated condensates via phase separation [9]. Interestingly, the role of each protein in forming the ePSD condensate is quite different. For example, in the receptor tail–PSD-95–GKAP–Shank–Homer organization, GKAP is a critical adaptor scaffold capable of connecting the receptor tail/PSD-95 sub-assembly with the Shank–Homer sub-assembly. Removal of GKAP leads to uncoupling of the two layers of sub-assemblies, and phosphorylation of GKAP can strengthen their coupling [74, 75]. In contrast, removing the molecules at either edge of the network (i.e. the receptor and Homer) only mildly reduced the enrichment of their direct binders into the ePSD condensates [9]. The layered structure of the ePSD condensates composed of many scaffold proteins may underlie the observations that the ePSD formation/dispersion can be regulated by alterations of different proteins in the ePSD and with broad dynamic ranges. It is noted that each of the major scaffold proteins shown in Fig. 2B contains multiple paralogs and each paralog further has multiple spliced isoforms. *In vitro* biochemical reconstitution experiments showed that ePSD condensates formed by different paralogs of scaffold proteins can display distinct properties. More interestingly, different isoforms of the same scaffold protein (e.g. Homer 1a vs Homer 1c) could have totally opposite roles in modulating ePSD condensate formation [9]. Thus, different neurons or even the same neurons at different developmental stages expressing different sets of scaffold proteins or their specific isoforms may form ePSDs with distinct molecular assemblies and functional features.

Recent high-resolution EM tomography studies revealed that the thin iPSD sheet is situated right beneath the transmembrane receptors. The electron densities of the protein particles within the iPSD sheet match the dimensions of a gephyrin E-domain dimer (i.e. ~11/5/5 nm in length/width/height) [24, 76]. Thus, the iPSD sheet is likely formed primarily by the single scaffold protein gephyrin. Given that other known iPSD proteins exist at much lower concentrations when compared to gephyrin, these proteins are probably either attached to the distal surface (relative to the synaptic membrane) of the iPSD sheet or exist within the iPSD sheet at sub-stoichiometric ratios. These proteins can play regulatory roles in modulating the iPSD formation and inhibitory receptor clustering. Because the formation of the iPSD condensates is chiefly determined by the binary interactions between the receptors and gephyrin, the broad diversity of inhibitory receptors, GABA_A_Rs in particular, may determine the assembly mechanisms and dynamic properties.

### Distinct dynamics between the ePSD and iPSD: appearance or occurrence

PSDs are dynamically modulated by synaptic activities, both structurally and functionally. As predicted by phase separation theory, there are two principal ways of modulating a biological condensate such as PSDs, either by altering the expression level of molecular components or by modifying their interactions in the condensates.

**Figure 3:**
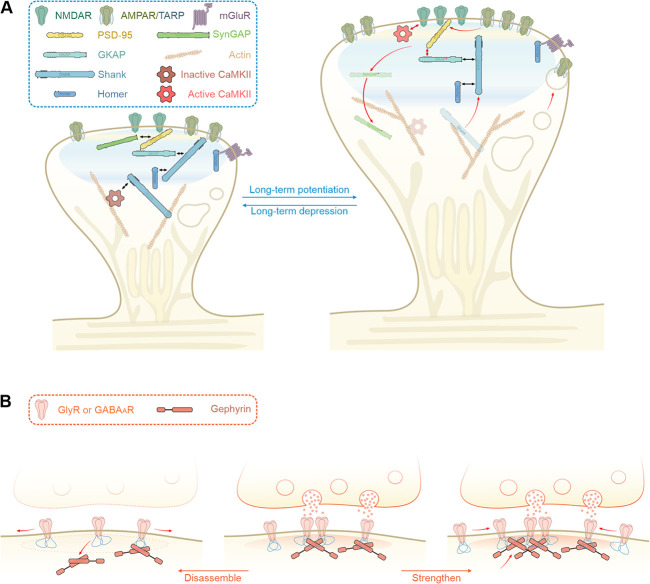
**Dynamic regulations of the iPSD and ePSD condensates.** (A) Schematic model showing activity-dependent regulation of the ePSD. During the synaptic activity, CaMKII-mediated phosphorylation and phosphatase-mediated dephosphorylation can bi-directionally modulate phase separation of the ePSD condensates, resulting in structural and functional changes of synapses. (B) Schematic model showing activity-dependent modulations of the iPSD during inhibitory synapse dynamics. The receptor-gephyrin interactions in the iPSD are critical for such regulation.

Both mechanisms appear to operate in the ePSD. It is well known that scaffold proteins as well as glutamate receptors can undergo activity-dependent exchange between pools in and outside the ePSD [77]. Overexpression or down-regulation of scaffold proteins are known to enlarge or shrink ePSDs, respectively. Upon stimulation, the thickness of ePSD gradually increases, accompanied by translocations of scaffold proteins to the ePSD [78]. Synaptic stimulation also activates various signaling pathways, resulting in modifications of protein–protein interactions among ePSD components and thereby modulating condensate formation or dispersion (Fig. 3A). For example, synaptic stimulation not only leads to activation of CaMKII but also can modulate the bindings of the enzyme to different components in the ePSD. Inactive CaMKII can bind to Shank3 [79], which is located near the pallium of the ePSD [78]. Upon stimulation, autophosphorylated CaMKII can translocate to the ePSD core through binding to GluN2B, forming CaMKII/GluN2B condensates [80]. The activated CaMKII can trigger further changes of the ePSD, for example, by phosphorylating and subsequent dissociating SynGAP from PSD-95 and by phosphorylating GKAP and promoting the coupling of the two sub-assemblies of ePSD discussed earlier above. Other enzymes, such as mitogen-activated protein kinase (MAPK), protein kinase A (PKA), protein kinase C (PKC) and its constitutively active isoform protein kinase M-zeta (PKMζ), have also been reported to modulate synaptic plasticity (see [81, 82] for reviews).

Neuronal activity-dependent posttranslational modifications also regulate receptor–gephyrin interactions and dynamics of the iPSD. However, rather than mainly affecting the size or shape of the PSD, synaptic and neuronal activities can directly regulate the formation of inhibitory synapses (Fig. 3B). For example, PKC-dependent phosphorylation in the GlyR-β subunit interferes with its high-affinity binding to gephyrin and block their phase separation [11], causing an increase in the GlyR diffusion in the plasma membrane and consequent reduction of iPSD clusters [83]. Similar events have been observed on postsynaptic GABA_A_R–gephyrin clusters as well. Interestingly, phosphorylation of gephyrin at different sites or at the same site but by various enzymes could have different impacts on the synaptic clustering of gephyrin and receptors. Studies on phosphorylation of residues Ser268 and Ser270 by ERKs and GSK3β, respectively, revealed a gephyrin turnover mechanism by calpain-dependent proteolysis for coordinated regulation of the cluster size and density on dendrites [84–86]. Whereas another research provided opposite observations when gephyrin serves as a CDK5 substrate [87]. Additionally, gephyrin clustering and synaptic transmission can be upregulated through phosphorylation of Ser305 of gephyrin by CaMKII [88], likely by releasing the autoinhibited conformation of gephyrin [11]. Other modifications, including palmitoylation, S-nitrosylation, acetylation and SUMOylation of gephyrin, may further regulate the iPSD-mediated receptor clustering [61]. In summary, unlike the ePSD regulated by multiple proteins in different layers of the PSD assembly, gephyrin is the master hub that can integrate many regulatory inputs to organize and modulate iPSD formation and receptor clustering.

Different molecular organization features of the ePSD and the iPSD are manifested by distinct dynamic properties of the two categories of PSDs. The ePSD network is more elaborate and inter-connected than the iPSD network (Fig. 2B vs D). Correspondingly, the ePSD is more stable than the iPSD. Compared to the iPSD, activity-dependent structural changes of the ePSD are reflected in the volume changes and molecule rearrangements, seldomly causing complete elimination of mature synapses. In contrast, the iPSD is much more dynamic and can be rapidly assembled or disassembled over a short-time window, likely because of the switch-like remodeling of the iPSD chiefly composed of only two proteins (i.e. the receptors and gephyrin). The ability to rapidly form or disperse is perhaps advantageous for inhibitory synapses to timely modulate balances of different neuronal circuits.

## SUBSYNAPTIC NANODOMAIN FORMATION AND TRANS-SYNAPTIC NANOCOLUMN ALIGNMENT

**Figure 4:**
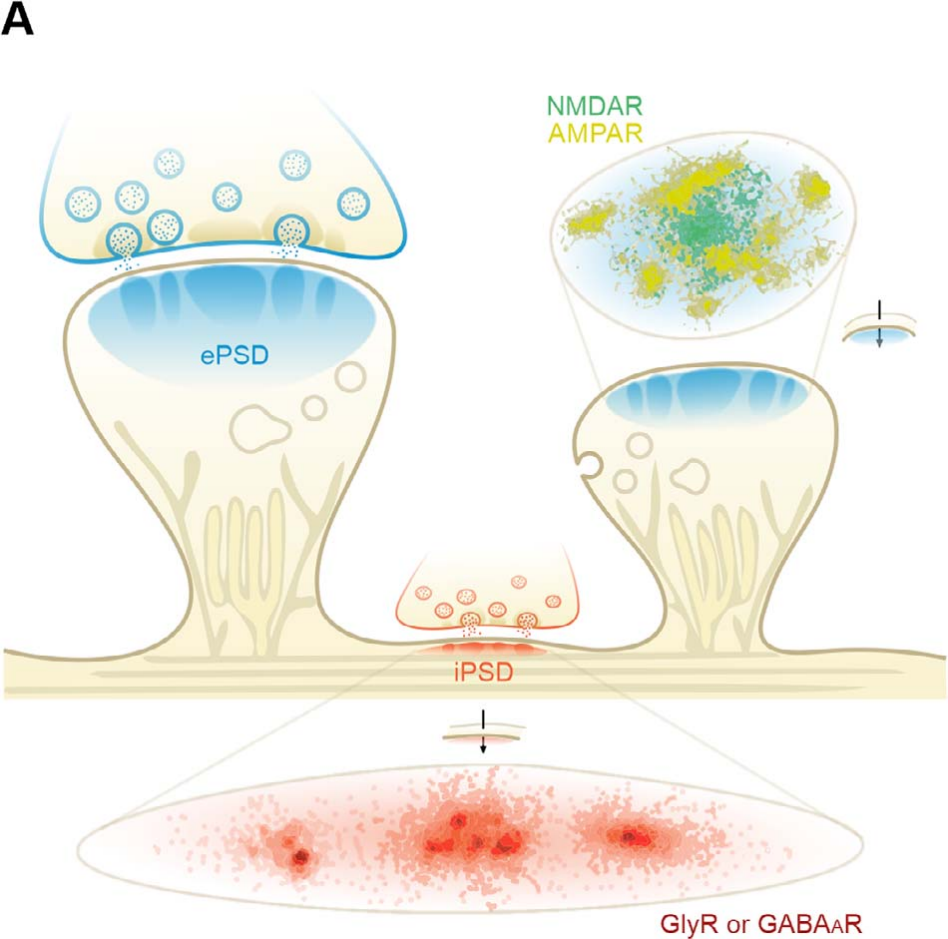
**Subsynaptic nanodomain formation and trans-synaptic nanocolumn alignment in excitatory and inhibitory synapses.** Presynaptic nanoclusters are closely aligned with nanodomains in postsynaptic sites forming trans-synaptic molecular nanocolumns. In the ePSD, the NMDAR cluster (in green) is surrounded by several AMPAR clusters (in yellow) visualized by dual-color dSTORM imaging. Adapted from [90]. The nanoscale arrangement of inhibitory receptors and gephyrin in the iPSD clusters is also drawn. Adapted from [101].

Recent advances in super-resolution microscopy techniques have revealed that receptors and scaffolds are further organized into subsynaptic domains (referred to as nanodomains) within PSDs (Fig. 4). In the ePSD, AMPARs are segregated into multiple subclusters at ~80 nm in diameter and presumably colocalized with PSD-95-containing nanodomains [89, 90]. Although NMDARs mainly display as a single cluster at the center of ePSDs, their subunit-dependent discrete nanodomain organization has also been reported [68]. Interestingly, NMDARs and AMPARs are kept apart in separate zones of the ePSD, with the NMDAR cluster at the center and several AMPAR nanodomains surrounding the NMDAR cluster. The formation of subsynaptic nanodomains is regulated by synaptic activities. Upon synaptic activation, more AMPARs are delivered to postsynaptic membranes, trapped by ePSD scaffolds and emerge as a new cluster(s). Conversely, synaptic depression is associated with nanodomain shrinkage and elimination. Similar observations were also made at inhibitory synapses showing that GlyRs or GABA_A_Rs were co-organized with gephyrin into nanodomains in the iPSD and rearranged during activity-dependent synaptic modulations [23, 24, 91]. Functionally, compared with crowding all the molecules in one cluster, distributing them into several nanoclusters may have advantages for the exchange of molecules between clusters and thus favor synaptic plasticity. Consistent with phase separation behaviors, proteins within PSD nanodomains are with limited mobilities, and proteins in less condensed regions but still within synapses are with somewhat higher mobilities. In contrast, proteins outside the PSD assembly (i.e. extrasynaptic proteins) undergo fast free diffusion motions.

Mechanisms governing subsynaptic nanodomain formation and co-segregation of nanodomains containing different receptors within one PSD are poorly understood. In theory, phase separation-mediated biomolecular condensate formation can provide explanations to such co-segregations of different nanodomains using so-called phase-in-phase and phase-to-phase arrangements of condensates [92, 93]. Such multiphase separation and co-segregation phenomena have recently been captured in an *in vitro* reconstituted ePSD system [80]. In that study, the authors showed that mixing GluN2B and TARP with PSD-95 resulted in formation of one homogenous phase with all three proteins coacervated. The addition of active CaMKII and subsequent phosphorylation of GluN2B led to persist segregation of the TARP-containing clusters from the GluN2B-containing clusters. Interestingly, NLGN1 was specifically co-segregated into the TARP-containing condensates through interacting with PSD-95. Thus, NLGN1 may play an important role in aligning the AMPAR nanodomain with the presynaptic active zone nanodomain that defines the neurotransmitter release site (see hereafter).

The multiphase organization typically occurs in multi-component systems such as the ePSD condensates. However, super-resolution imaging studies have observed the formation of nanodomain-like organizations in the iPSD [91]. At the current stage, it is not understood how such nanodomain structure forms in the iPSD and what proteins, in addition to the neurotransmitter receptors and gephyrin, may be needed for the iPSD nanodomain formation. The nanoscale iPSD clusters may also reflect a size-control mechanism of phase separation driven by the magic-number effect [94]. For strongly associated systems, especially in two-component systems, the growing of phase-separated condensates is suppressed at certain stoichiometries, favoring the formation of small stable nanoclusters. In line with this theoretical study, it was recently observed that, during inhibitory synapse growth, the iPSD enlargement (or shrinkage) is accompanied by an increase (or decrease) in the number of gephyrin nanodomains rather than simply enlarging (shrinking) the existing iPSD condensates [91, 95]. A recent EM tomography study revealed a mesophasic organization of GABA_A_Rs in synapses by a high frequency of ‘linked’ receptor pairs or triplets in the iPSD [24]. Biochemical reconstitution studies showed that the droplets of the receptor–gephyrin complex condensates were small and did not proportionally grow when concentrations of the proteins increase [11].

In the presynaptic compartment that is directly juxtaposed to PSDs, synaptic vesicles are tethered to active zones, which are specialized structures exhibiting discrete dense projections under EM and are also shown to be formed via phase separation [15, 17]. Importantly, the presynaptic RIM nanodomain is aligned with the postsynaptic nanodomains containing PSD-95-clustered AMPARs, forming trans-synaptic molecular nanocolumns [96]. Formation of the trans-synaptic nanocolumns may position clustered neurotransmitter receptors precisely in proximity to vesicle release sites, thus allowing fast and precisely timed synaptic responses. The trans-synaptic nanocolumn formation is likely to be critical for AMPARs- and GABA_A_Rs-mediated synaptic transmissions as the receptors bind to their respective neurotransmitters (glutamate and GABA, respectively) with very low affinities. Many adhesion molecules have been proposed to orchestrate such trans-synaptic nanocolumn alignment. For example, deletion or mutation of adhesion proteins such as NLGNs and ADAM22 led to impairments of trans-synaptic alignment and dysfunctions of synaptic transmissions [97, 98]. It should be noted that the formation of nanodomains in each side of synapses with cell adhesion molecules altered were still intact, suggesting that these cell adhesion molecules do not instruct the formation of nanodomains in both presynaptic active zones and PSDs.

## CROSSTALK BETWEEN THE EPSD AND IPSD

The tight control of the number and distribution of excitatory and inhibitory synapses is essential for neurons and neuronal circuits to function properly. Excitatory synaptic inputs can modulate local dendritic inhibitory synapse formation. Inhibitory synapses are in return essential for integrating excitatory synaptic events and regulating synaptic plasticity. Most inhibitory synapses are located in dendritic shafts where local computations are performed within specific dendritic branches. There are also high-density inhibitory synapses on cell soma and axon initial segments, synchronously influencing overall neuronal firing (Fig. 1A). A small portion of inhibitory synapses forms on dendritic spine protrusions where excitatory synapses also form (Fig. 1B). Such co-innervation of an inhibitory synapse and an excitatory synapse on the same dendritic spine may fine-tune individual spine conductance and excitation [99]. Interestingly, even though excitatory and inhibitory PSDs coexist within the same micron-sized postsynaptic compartment, the two assemblies with opposite electric functions should not overlap and have indeed been shown to be separated by EM studies [99]. Little is known regarding the mechanism underlying segregation of the ePSD and iPSD condensates within such tiny postsynaptic compartments. On the other hand, although the ePSD and the iPSD are formed by a distinct set of proteins, the two PSD assemblies do share some signaling components such as Ca^2+^, CaMKII and other signaling molecules. Thus, the ePSD and iPSD condensates in synapses can communicate through these shared molecules [100]. Studying how the ePSD and iPSD communicate with each other is a fertile future research ground.

## CONCLUSIONS

Synaptic transmission is conducted by pre- and post-synaptic specialized machineries, each formed by a unique set of densely packed proteins via phase separation. Extensive studies in the past few decades have generated a wealth amount of knowledge on how the ePSD is formed and regulated. In contrast, even though compositionally simpler than the ePSD, our understanding of iPSD formation and regulation is not as advanced. Nonetheless, recent biochemical and biophysical studies, including but not limited to proteomic characterization of the iPSD proteome, super-resolution imaging studies of the dynamic properties of iPSD clusters, *in vitro* reconstitution of the iPSD condensates, etc., have significantly advanced our understanding of the iPSD. These studies have also opened avenues for better understanding the organization principles of the iPSD and the underlying mechanisms governing the functions of inhibitory synapses. Both the diversity of the molecular compositions of inhibitory neurotransmitter receptors and the highly dynamic nature of the iPSD assemblies impose technical challenges in investigating the structure and functions of the iPSDs. The development of new technologies in studying the iPSD at higher resolution in living neurons will advance the field.

Box 1: Key questions to be answered for better understanding of the iPSD formation and regulation

1. Recent advances in microscopy and proteomic studies have advanced our understanding of the functional architecture and organization principles of the iPSD. However, inhibitory synapses are highly diverse. Neuronal type-specific or even single-synaptic level proteomic analysis will be extremely valuable for understanding different types of iPSD formation and regulation.

2. New iPSD proteins, such as InSyn1 and InSyn2, are continuously being identified, but functional studies of these proteins trail behind. Future studies are needed to characterize roles of these proteins in iPSD formation and plasticity.

3. Molecular mechanisms underlying subsynaptic nanodomain formation for both the iPSD and ePSD are still poorly understood. Whether the formation of subsynaptic nanodomains in the iPSD requires other proteins and if yes how these proteins modulate the iPSD nanodomain formation are interesting topics for future studies.

4. What are the molecular mechanisms governing the segregation between the iPSD and the ePSD? How the two PSD condensates communicate with each other?

5. The interplay between PSDs and presynaptic molecular assemblies through trans-synaptic adhesion proteins is another fascinating area of future research.

6. Why and how mutations of genes encoding key PSD proteins may perturb PSD assembly formation and regulation? This is an important area of research for understanding broad spectrum of brain disorders caused by mutations of genes encoding synaptic proteins.

## SUPPLEMENTARY MATERIAL

Supplementary material are available at *Oxford Open Neuroscience* online.

## CONFLICT OF INTEREST

None declared.

## AUTHORS’ CONTRIBUTIONS

G.B. and M.Z. conserved the idea and wrote and revised the manuscript.

## Supplementary Material

suppl_data_kvac003
