# Supplementary material for: Inhibitory postsynaptic density from the lens of phase separation
Source: Oxf Open Neurosci. 2022 May 4;1:kvac003. doi: 10.1093/oons/kvac003 (PMC10913824; doi:10.1093/oons/kvac003)
Supplement: suppl_data_kvac003 [file suppl_data_kvac003.zip › kvac003_REVIEWER COMMENTS oxfnsc-2022-002.docx]

REVIEWER COMMENTS

OXFNSC-2022-002 Inhibitory postsynaptic density from the lens of phase separation

Original submission

Reviewer 1 comments

Review of “Inhibitory postsynaptic density from the lens of phase separation” by Carlos Ibanez

This is a timely, interesting, and well-written piece summarizing recent discoveries relating to the structure, organization and function of the post-synaptic density (PSD), with emphasis on the less well-known inhibitory PSD. The different topics (molecular components, scaffolding, phase separation mechanisms, etc.) are logically and clearly presented. The text flows nicely and it is easy to read and understand. Perhaps the authors would like to consider adding a Box with bullet points highlighting the most important problems for future research in this area. It would be useful to have these summarized in one distinct place, in addition to presenting them along the relevant sections in the text. On minor comment, on page 13, end of line 35, I think it should read “may have” rather than “may be”.

Reviewer 2 comments

This is a very nice review describing the role of excitatory and inhibitory components of neuronal components. More specifically the authors provided detailed overview of the composition, organizational and regulatory features of inhibitory post synaptic densities in the angle of phase separation concept. 
I have the following minor suggestions for improvement:
1. Currently the review is focused more on molecular aspects of ePSDs and iPSDs. It will be useful for the broad audience if the authors can provide a brief introduction about the functional plasticity mediated by ePSDs and iPSDs in general.
2. Currently ionotropic receptors (AMPA and NMDA) are represented in the Fig.3A. The authors should update the figure by including mGluRs, which plays an important role in the signal transduction process that leads to long-term plasticity.
3. In Fig.3A, the arrow representing synaptic plasticity is bidirectional. Please provide example for the type of activity. For instance, LTP or LTD?
4. The role of CaMKII is explained well but there are also other molecules which drive the AMPAR subunits to PSD. For instance, PKMzeta.

Editor decision letter

|  | 15-Feb-2022   Dear Dr. Zhang,   Manuscript ID OXFNSC-2022-002 entitled "Inhibitory postsynaptic density from the lens of phase separation" which you submitted to the Oxford Open Neuroscience, has been reviewed by myself and an anonymous reviewer.  The comments are included at the bottom of this letter.   We are happy to accept this manuscript pending minor revisions as suggested in the comments, which I hope you find useful    To revise your manuscript, log into https://mc.manuscriptcentral.com/oxfnsc and enter your Author Centre, where you will find your manuscript title listed under "Manuscripts with Decisions."  Under "Actions," click on "Create a Revision."  Your manuscript number has been appended to denote a revision.   You may also click the below link to start the revision process (or continue the process if you have already started your revision) for your manuscript. If you use the below link you will not be required to login to ScholarOne Manuscripts.   *** PLEASE NOTE: This is a two-step process. After clicking on the link, you will be directed to a webpage to confirm. ***   https://mc.manuscriptcentral.com/oxfnsc?URL_MASK=777167403f5844eebe9ecc9aa26a718a   Once the revised manuscript is prepared, you can upload it and submit it through your Author Centre. When submitting your revised manuscript, you will be able to respond to the comments made by the reviewer(s) in the space provided.  You can use this space to document any changes you make to the original manuscript.  In order to expedite the processing of the revised manuscript, please be as specific as possible in your response to the reviewer(s).   IMPORTANT:  Your original files are available to you when you upload your revised manuscript.  Please delete any redundant files before completing the submission.   Once again, thank you for submitting your manuscript to the Oxford Open Neuroscience and I look forward to receiving your revision.   Sincerely,  Carlos Ibanez  Senior Editor, Oxford Open Neuroscience  carlos.ibanez@pku.edu.cn    Authors response  Feb. 21, 2022  Carlos Ibanez  Senior Editor, Oxford Open Neuroscience   Re: Manuscript ID OXFNSC-2022-002   Dear Carlos,  Thank you so much for your editorial letter regarding our review manuscript entitled “Inhibitory postsynaptic density from the lens of phase separation”. We have revised the manuscript following comments and suggestions from you and the other reviewer. Our point-by-point responses to the reviewers’ comments are at the end of this letter. Hope that our revisions are satisfactory.  I am looking forward to successful development of the Oxford Open Neuroscience.   With best regards,    Responses to the comments   Reviewer: 1   This is a timely, interesting, and well-written piece summarizing recent discoveries relating to the structure, organization and function of the post-synaptic density (PSD), with emphasis on the less well-known inhibitory PSD. The different topics (molecular components, scaffolding, phase separation mechanisms, etc.) are logically and clearly presented. The text flows nicely and it is easy to read and understand. Perhaps the authors would like to consider adding a Box with bullet points highlighting the most important problems for future research in this area. It would be useful to have these summarized in one distinct place, in addition to presenting them along the relevant sections in the text. On minor comment, on page 13, end of line 35, I think it should read “may have” rather than “may be”.   Thanks so much for the warm comments and wonderful suggestion. Following your suggestion, we have added a Box highlighting some of the key questions to be answered for better understanding of the iPSD formation and regulation in the revised manuscript. We have also fixed the language error that you have spotted.    Reviewer: 2   This is a very nice review describing the role of excitatory and inhibitory components of neuronal components. More specifically the authors provided detailed overview of the composition, organizational and regulatory features of inhibitory post synaptic densities in the angle of phase separation concept.  We thank the reviewer for the enthusiastic comments and great suggestions. We have revised the manuscript following the reviewer’s suggestions (see below).   I have the following minor suggestions for improvement:  1. Currently the review is focused more on molecular aspects of ePSDs and iPSDs. It will be useful for the broad audience if the authors can provide a brief introduction about the functional plasticity mediated by ePSDs and iPSDs in general.  It is a wonderful suggestion. We have added a few sentences introducing the functional plasticity mediated by ePSDs and iPSDs in the “Introduction” section of the revised manuscript (lines 14-19 of the section).   2. Currently ionotropic receptors (AMPA and NMDA) are represented in the Fig.3A. The authors should update the figure by including mGluRs, which plays an important role in the signal transduction process that leads to long-term plasticity.  Thanks for the suggestion! We have added mGluRs in the revised Fig. 3A.   3. In Fig.3A, the arrow representing synaptic plasticity is bidirectional. Please provide example for the type of activity. For instance, LTP or LTD?  We have modified Fig. 3A arrow to be directional. Since this review is focusing on iPSD, we prefer not to expand examples of LTP and LTD for ePSD in order not to shift the focus. Additionally, this manuscript is already a bit too long.   4. The role of CaMKII is explained well but there are also other molecules which drive the AMPAR subunits to PSD. For instance, PKMzeta.  Following the reviewer’s suggestion, we have added that other enzymes including PKMζ, PKC, mitogen-activated protein kinase (MAPK), and protein kinase A (PKA) have also been reported to modulate synaptic plasticity in the revised manuscript (end of the first paragraph on page 12). |
| --- | --- |

Revised submission

Immediate accept on revision no further AE comments.

Decision letter

| **Subject:** | Oxford Open Neuroscience - Decision on Manuscript ID OXFNSC-2022-002.R1 |
| --- | --- |
| **Body:** | 22-Feb-2022   Dear Mingjie,   It is a pleasure to accept your revised manuscript entitled "Inhibitory postsynaptic density from the lens of phase separation" in its current form for publication in the Oxford Open Neuroscience. Many thanks for your contribution.  On behalf of the Editors of the Oxford Open Neuroscience, we look forward to your continued contributions to the Journal.   Best regards,  Carlos |
